# Micro- CT evaluation of sealers removal by reciprocal instrumentation followed by continuous ultrasonic irrigation in teeth with oval root canals

**DOI:** 10.4317/jced.60013

**Published:** 2023-03-01

**Authors:** Janaina-Araujo Colombo, Daniel-Guimarães-Pedro Rocha, Ana-Grasiela-da Silva Limoeiro, Wayne-Martins Nascimento, Carlos-Eduardo Fontana, Rina-Andrea Pelegrine, Carlos-Eduardo-da Silveira Bueno, Alexandre-Sigrist De Martin

**Affiliations:** 1 Faculdade São Leopoldo Mandic, Instituto de Pesquisas São Leopoldo Mandic, Endodontia, Capinas, SP, Brazil; 2 Pontifícia Universidade Católica de Campinas, Centro de Ciências da Vida, Programa de Pós-graduação em Ciências da Saúde, Campinas, SP, Brazil

## Abstract

**Background:**

The ability of the Reciproc system (R40) followed by continuous ultrasonic irrigation (CUI) to remove filling material from oval root canals of mandibular premolars filled with gutta-percha and AH Plus or Bio-C Sealer was evaluated by microtomography (micro-CT).

**Material and Methods:**

The straight and oval root canals of 42 mandibular premolars were prepared with the ProDesign R 35.05 reciprocal file and randomly divided into two groups according to the material used to fill the canals (n=21): Group AH - Master Cone and AH Plus; Group BC - Master Cone and Bio-C Sealer. After filling and provisional sealing, the teeth were stored at 100% relative humidity and a temperature of 37°C for 30 days. The filling material was then removed with an R40 file. The material was considered completely removed when the R40 file reached the working length (WL), and no remaining filling material was visible on the canal walls. CUI was then performed. The teeth were scanned by micro- CT before and after removal of the filling material. The remaining filling material was measured in mm in the last apical 5 mm. The data were analyzed with the nonparametric Friedman test and subsequently with the Dunn test. The Mann-Whitney U test was also performed. Statistical significance was accepted at the 5% level.

**Results:**

After instrumentation with the Reciproc R40, the volume of residual filling material was significantly greater in the BC group than in the AH group (*P* = 0.035). After CUI, there was no difference in the volume of residual material between the two groups (*P* = 0.705).

**Conclusions:**

Bio-C sealer was more difficult to remove with the Reciproc file than AH Plus. CUI improved the removal of residual filling material regardless of sealer type. However, no technique was able to completely clear the canals of filling material.

** Key words:**Bioceramic cement, CUI, micro-CT, reciproc, retreatment.

## Introduction

The success of endodontic therapy depends on the removing necrotic material, inflamed tissue, microorganisms, and debris from the root canal system and preventing subsequent recontamination of the canals to create an environment conducive to repair (1-4). The persistence of bacteria in the root canal system is the main cause of disease after endodontic treatment. In these cases, conventional endodontic retreatment is indicated as the first treatment option of choice.

Various techniques have been tested to remove the filling material (gutta-percha and sealer) from the canal for retreatment, including the use of manual, rotary, and reciprocal instruments. However, to date, none of the techniques evaluated have been able to completely remove these materials from the root canal system (5-11).

In view of this, there is a need for additional methods to assist in the removal of filling material from canals for retreatment, such as sonic or ultrasonic activation of the irrigation solution, which allows for more effective cleaning of the root canal system (12-16); these may also be influenced by the type of sealer used in the first round of endodontic treatment.

In continuous ultrasonic irrigation (CUI), the irrigation solution is simultaneously exchanged and activated. The main advantage of this technique is the continuous supply of fresh irrigant, which improves the removal of contents from the root canal system, while passive ultrasonic irrigation (PUI) requires manual replacement of the irrigant between activations.

The Irrisonic E1 (Helse, Santa Rosa do Viterbo, Brazil) ultrasonic insert features a special stainless design and alloy. It has no cutting blades and can be pre-curved at its end, just as a stainless-steel file is pre-curved. High ability to promote microacoustic flow. Its diameter is the same as a #20 hand file with a 0.01 taper and should be used up to 1 mm below the WL at 10% power, according to the manufacturer.

Micro-CT has been used to evaluate the effectiveness of various techniques for removing filling material from root canals. This nondestructive method provides an accurate, quantitative, and three-dimensional evaluation of the remaining filling material (2,6,17). By allowing observation of the canal during the different stages of retreatment, micro- CT overcomes the limitations of the other assessment methods (18).

AH Plus (AH - Dentsply Sirona, Konstanz, Germany) is an epoxy resin-based endodontic sealer that is widely used due to its good sealing ability, adhesion to the root dentin, and low solubility. It has adequate radiopacity and shows sufficient adaptation to the root canal wall and penetration into the dentinal tubules. However, its major limitation is the lack of bioactive properties (19).

Bio-C Sealer (BC - Angelus Indústria de Produtos Odontológicas S/A, Londrina, Paraná, Brazil) is a bioceramic endodontic sealer composed of calcium silicate, calcium aluminate, calcium oxide, zirconium, iron oxide, silicon dioxide, and dispersants. According to the manufacturer, this sealer has biocompatibility, bioactivity, and high pH, radiopacity, and flow values. However, there is limited research on the extent to which this sealer can be removed unlike AH.

It is important to understand whether these novel ready-to-use calcium silicate-based sealers can be removed, especially as they are routinely used in clinical practice and in cases where retreatment may be required (20). This study investigated the ability of the Reciproc system (R40) followed by CUI to remove filling material from oval root canals of mandibular premolars filled with gutta-percha and AH or BC. The null hypotheses tested were that there is no difference in the removal of the two filling materials when the R40 is used and that the use of CUI does not increase the removal of these filling materials.

Material and methods

This study was approved by the local institutional research ethics committee (protocol no. 3.017.368). Forty-two single-rooted human mandibular premolars were selected. Only teeth with a single oval root canal (buccolingual diameter twice the mesiodistal diameter, measured 5 mm from the root apex), a fully formed apex, and a single foramen were included. The presence of these features was verified by buccolingual and mesiodistal radiographs. Roots with a curvature of more than 20° according to the method of Schneider’(21) were excluded. Considering a statistical power of 80%, an error rate of 5%, and an effect size of 0.85, the minimum sample size was set at 18 samples per group. To account for possible losses that might occur during the experiment, 42 specimens (n=21) were used.

-Preparation of the specimens

Crowns were removed with a double-sided diamond disk (KG Sorensen 7020, Cotia, Brazil) and roots were standardized to a length of 16 mm (Mitutoyo Absolute Vernier Digital Caliper, capacity 150 mm, Mitutoyo Sul Americana Ltda., Suzano, Brazil). Patency was confirmed with a #10 K-file (Dentsply Sirona, Konstanz, Germany) 1 mm anterior to the anatomic apex. The WL was determined for each tooth 1 mm anterior to the point where the #10 K-file crossed the apex under 8x magnification (DFV Vasconcellos, Valença, Rio de Janeiro, Brazil.

Biomechanical preparation was performed by a single operator using a VDW Silver endo-motor (VDW GmbH, Munich, Germany) in Reciproc-All mode in a small amplitude pecking motion. Two reciprocal NiTi ProDesign R files (25.06 + 35.05, Bassi/Easy Equipamentos Odontológicos, Belo Horizonte, Brazil) were used for 3 teeth in each group, for a total of 7 ProDesign R25 and R35 files per group. These files have a double helix cross-section and are heat-treated with CM. The instruments were moved with light pressure movements with an amplitude of approximately 3 mm in the apical direction. After three pecking motions, the instruments were removed from the canal and carefully cleaned with an alcohol pad. This protocol was repeated until the instruments reached WL. A constant irrigation with 10 mL of sodium hypochlorite (NaOCl) 2.5% (ASFER Indústria Química Ltda, São Caetano do Sul, Brazil) per tooth was performed. A final irrigation was performed with 5 mL EDTA 17% (Biodinâmica Química e Farmacêutica Ltda, Ibiporã, Brazil) followed by 5 mL NaOCl 2.5%. PUI was not performed at this stage of treatment since the objective of the study was to evaluate the effect of CUI on retreatment.

-Obturation

The specimens were randomly divided (www.random.org) into two groups: AH (AH Plus) and BC (Bio-C Sealer).

AH group: teeth were dried with calibrated 35.05 absorbent paper cones (Cell Pack Easy, Tanari) and filled with AH and 35.05 master cones (Gutta Percha Calibrated Tip Endo Tanari Plus). AH was prepared according to the manufacturer’s instructions. Three parts of component A and three parts of component B were mixed on a glass plate with a #24 spatula in a circular motion. The sealer was inserted into Bio-C sealer silicone tips and injected with a 3-mL Luer-lock disposable syringe (BD Indústria Cirúrgica S/A, Juiz de Fora, Brazil) until the sealer was visible in the coronal portion of the specimens. One tip was used for each group of 3 teeth. The preselected master cone was inserted, and excess was cut off with a heat plugger. Vertical condensation was performed. The teeth were sealed with Coltosol (Coltene, Vigodent SA Indústria e Comércio Ltda., Rio de Janeiro, Brazil), and mesiodistal and buccolingual radiographs were taken to assess the quality of obturation.

BC group: the same absorbent paper cones and Gutta Percha master cones were used, but BC sealer was used instead of AH. The ready-to-use sealer was injected into the silicone tips according to the manufacturer’s guidelines. As in the AH group, the sealer was injected until it was visible in the coronal area of the specimens. Again, one tip was used for each group of 3 teeth. All specimens from both groups were sealed with Coltosol, immersed in distilled water (ASFER Indústria Química Ltda., São Caetano do Sul, Brazil) and stored for 30 days at a constant temperature of 37 °C and relative humidity of 100%.

-Micro- CT Analysis

After 30 days, the first scan was performed with a Skyscan 1173 micro- CT scanner (Bruker Micro CT, Kontich, Belgium) to assess the quality of obturation and to measure the total volume of filling material (in mm³) in the AH and BC groups. The Skyscan 1173 uses a cone beam X-ray geometry for image acquisition. During data acquisition, the object can be rotated 180° or 360° in fixed steps. An image is captured at each step. The scans were saved as 16-bit files in Tag Image File Format (TIFF). The scanner operates in a voltage range of 30 to 130 kV, with variable current, up to a maximum power of 8 W. After the acquisition process, the captured image is reconstructed using an FDK algorithm. Data acquisition is performed using Skyscan 1173 software, while image reconstruction is performed using NRecon, version 1.6.9.4. Qualitative and quantitative analyzes were performed in CTVox version 3.0.0r1114 and CTAn version 1.16.4.1, respectively. One reviewer analyzed all images. Samples were scanned with optimal voltage and current parameters: Energy 70 kV, current 114 µA and pixel size 8.5 µm.

-Retreatment

After the first micro- CT scan of both groups, the filling material was removed with a Reciproc R40 file (VDW GmbH, Germany) and a #10 K-file for 3 teeth in each group, always using a VDW Silver Endo motor in Reciproc All mode, with short pecking motions and brushing against the lateral walls until the WL was reached. No solvents were used. All procedures were performed by the same operator. Filling material removal was considered complete when the WL was reached, and no residual filling material was visible on the file. Likewise, when apical patency was achieved with the #10 file. The irrigation solution (sodium hypochlorite 2.5%) was refreshed at each step. The final rinse consisted of 5 mL EDTA 17% + 5 mL sodium hypochlorite 2.5%, and specimens were sealed with Coltosol. A second scan was performed to assess the amount of remaining filling material (in mm³) in the AH and BC groups after instrumentation with the R40 file.

Two teeth in the AH group experienced a fracture of the apical third during the removal of the filling material; both were excluded from further analysis.

-Continuous ultrasonic irrigation (CUI)

All remaining specimens (19 teeth from the AH group and 21 teeth from the BC group) still showed residual filling material in the apical 5 mm at the second micro- CT analysis. Therefore, a CUI was performed. The rinse agent of choice for this step was distilled water, as the goal was to evaluate the effect of CUI without any interference from NaOCl. An Irrisonic E1 insert was used in a Satelec ultrasonic unit (Acteon Micro Imagem Indaiatuba, Brazil) set at 10% power. Distilled water was continuously agitated for 1 minute with small amplitude movements of 1 mm up of WL. After CUI, a third scan was performed to evaluate the volume of remaining filling material in the AH and BC groups.

-Statistical analysis

Statistical analyzes were performed using SigmaPlot version 12.5 software. The significance level was set at 5%. The nonparametric Friedman test was used (*P* < 0.001), followed by Dunn’s post hoc test (*P* < 0.05). The Mann-Whitney U test was also performed (*P* < 0.05).

## Results

The remaining filling material was measured after using R40 and after the use of R40 and CUI. When the R40 file was used, the volume of filling material removed in the apical 5 mm was significant in both groups compared with the baseline volume (*P* < 0.001). After instrumentation with the R40 file, a larger residual volume was found in the BC group than in the AH group (*P*=0.035).

**Figure 1:**
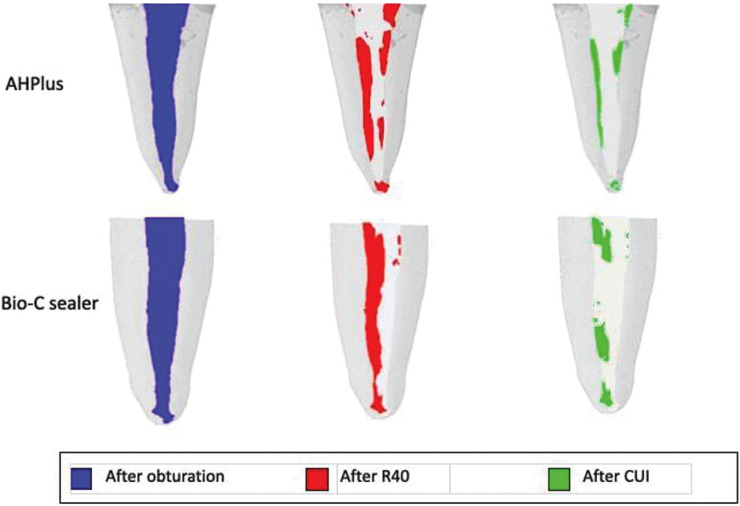
Views of representative micro- CT reconstructions of the root canals, showing the remaining filling material before (blue), after the use of R40 (red) and after the use of CUI (green).

CUI significantly decreased the volume of residual filling material in both groups (*P* < 0.001). Nevertheless, there was no significant difference between the AH and BC groups in the volume of remaining material after instrumentation with the R40 file plus CUI (Mann-Whitney U, *P*=0.159 for the first stage; *P*=0.705 for the ultrasonic stage). The volume of filling material remaining in the teeth in the AH and BC groups at each stage of retreatment, is shown in Table 1 and Figure 1.

**Table 1:**
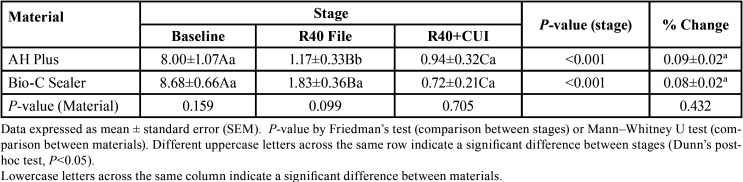
Volume of material (AH Plus cement vs. Bio-C Sealer) remaining in the tooth at each stage of retreatment and percent variation from baseline (filling) to instrumentation with R40 file and CUI. Values expressed as mm3.

## Discussion

This study investigated the efficacy of instrumentation followed by CUI in removing gutta-percha and two different types of obturation material: Bio-C Sealer and AH Plus, an from mandibular premolars with oval-shaped root canals. The null hypothesis was partially accepted because there was no difference between the groups in terms of CUI. However, there were differences in terms of filling material removal with the R40 file.

One of the characteristics of an ideal sealer is that it can be removed from the root canal system when needed. The degree of adhesion of the sealer to the dentin walls depends on the quality of dentin cleaning prior to obturation (20). This is an important factor in the proper seating of the sealer, which can be difficult to achieve in the apical region and at the isthmus due to difficulties in removing debris in these areas (17,22). Eymirli *et al*. (20) found that EndoSequence BC Sealer was impermeable to manual instruments in the apical foramen. In the present study, such an observation was not made because the canals were filled not only with sealer but also with gutta-percha and because the tooth was selected for analysis.

Previous studies have shown that the use of solvent can produce a “slurry” of filling material that can penetrate into isthmuses, lateral canals, and irregular features of the root canal system. This substance can make complete cleaning of the root canal system difficult or even impossible and is also cytotoxic to periapical tissue. Another aspect that has been demonstrated is the large capacity of rotary and reciprocal instruments to remove filling material (6,14). These aspects justify the decision not to use solvents in the present study.

The larger grooves and S-shaped cross-section of the Reciproc instrument allowed greater removal of dentin and/or filling material with less extrusion of debris. The reciprocating motion has a counterclockwise cutting action and a clockwise “unscrewing” force (150° counterclockwise and 30° clockwise at 10 cycles per second in a VDW motor, equivalent to 300 rpm).

AH contains a polymer that contracts after polymerization, which can cause seal failure and deterioration. Therefore, it should be easier to remove, even if only partially. Bioceramics, on the other hand, should be more difficult to remove due to their strong bond to dentin and promotion of hydroxyapatite formation. It is likely that BC in the present study had a larger residual volume for these reasons. Similar results were reported by Huang *et al*. (17) and Romeiro *et al*. (11). In contrast, Bago *et al*. (23) claimed that it proved more difficult to completely remove epoxy-based sealer compared with bioceramics in retreatment cases. Suk *et al*. (24) conducted a study using phototherapy and found no differences between epoxy resin and bioceramic cements in retreatment cases. These differences can be explained, at least in part, by differences in methodology between the studies.

The results of the present study, which are consistent with those of Bernardes *et al*. (8) and Zuolo *et al*. (5), suggest that AH and BC leave similar residual volumes in the root canal system after both removal levels tested in this experiment. CUI reduced the residual volume of filling material in both the AH and BC groups, even without the use of solvents; however, it was unable to completely remove either sealer from the oval-shaped canals. Other studies have reported similar results regarding the efficacy of irrigation methods in retreatment cases (2,14-16,25,26).

In agreement with previous studies, we conclude that conventional retreatment techniques are not able to completely remove endodontic sealers, including bioceramics (2,8,13-16,27-29). The low residual volume of filling material in the samples measured by micro- CT highlights the good performance of the tested instrument, although it was not able to completely remove the material (30).

Therefore, additional methods are needed to help remove residual filling material from inside the root canal system in preparation for retreatment. The continuous ultrasonic movement of the irrigation solution improved the cleaning of the root canal system.

## Conclusions

It was found that Bio-C sealer was more difficult to remove with Reciproc R40 instrumentation than AH Plus. The CUI improved the removal of the remaining filling material regardless of the cement type, but still could not remove it completely.
